# A spatially-explicit harmonized global dataset of critical infrastructure

**DOI:** 10.1038/s41597-022-01218-4

**Published:** 2022-04-01

**Authors:** Sadhana Nirandjan, Elco E. Koks, Philip J. Ward, Jeroen C. J. H. Aerts

**Affiliations:** 1 Institute for Environmental Studies (IVM), Vrije Universiteit Amsterdam, 1081HV Amsterdam, the Netherlands; 2 Environmental Change Institute, University of Oxford, Oxford, OX1 3QY United Kingdom

**Keywords:** Natural hazards, Databases, Energy access

## Abstract

Critical infrastructure (CI) is fundamental for the functioning of a society and forms the backbone for socio-economic development. Natural and human-made threats, however, pose a major risk to CI. Therefore, geospatial data on the location of CI are fundamental for in-depth risk analyses, which are required to inform policy decisions aiming to reduce risk. We present a first-of-its-kind globally harmonized spatial dataset for the representation of CI. In this study, we: (1) collect and harmonize detailed geospatial data of the world’s main CI systems into a single geospatial database; and (2) develop the Critical Infrastructure Spatial Index (CISI) to express the global spatial intensity of CI. The CISI aggregates high-resolution geospatial OpenStreetMap (OSM) data of 39 CI types that are categorized under seven overarching CI systems. The detailed geospatial data are rasterized into a harmonized and consistent dataset with a resolution of 0.10 × 0.10 and 0.25 × 0.25 degrees. The dataset can be applied to explore the current landscape of CI, identify CI hotspots, and as exposure input for large-scale risk assessments.

## Abstract (summary)

**Table:** 

Measurement(s)	geospatial location of global critical infrastructure • Critical Infrastructure Spatial Index • critical infrastructure density at the global scale
Technology Type(s)	Volunteered Geographic Information • spatial analysis
Factor Type(s)	high-detailed geospatial data
Sample Characteristic - Organism	Critical infrastructure
Sample Characteristic - Environment	Built environment
Sample Characteristic - Location	Global

## Background & Summary

Critical infrastructure (CI) plays a crucial role in the delivery of services that are vital for the functioning of our society, from the provision of reliable energy services to telecommunication^1,2^. Interruptions in the service provision of CI can occur due to disruptive events which are a direct cause of socio-economic disruptions^3^. A disruptive event can be both natural (e.g. earthquakes, floods, and tropical storms) or anthropogenic (e.g. poor maintenance, mismanagement, and terrorist attacks) in origin^4,5^. The impacts of such disruptive events on CI can harm society directly and indirectly. Firstly, they may cause direct physical damages to CI assets^6^. Secondly, due to the (inter)dependent nature of infrastructure systems, a causal chain of disruptions may occur between different infrastructure systems^7^. Thirdly, these impacts may translate into far-reaching indirect repercussions across different regions or sectors as a result of the large dependency of society to CI^8–12^. For example, the disruption of services costs firms and households in low- and middle- income countries at least $390 billion a year, while the indirect impacts further aggravate the burden on society^1^.

Natural hazards are a large source of potential damage, and therefore pose a major threat to CI^1,2,13,14^. Climate change and the associated intensification and increased frequency of hazards will increase the impacts of natural hazards on CI^13,15–17^, while socio-economic development will lead to an increase in the amount and value of CI exposed to hazards. Given these challenges it is no surprise that *Building resilient infrastructure* is an explicit part of Goal 9 of the Sustainable Development Goals (SDGs)^18^, with the aim of reducing the risks of natural hazards and climate change on society^1,19–21^. This is supported by the UN Sendai Framework for Disaster Risk Reduction (SFDRR)^22^, which calls for assessing CI risk, and the development of open access databases on CI exposure to support risk assessment.

However, only a limited number of studies have examined the spatial patterns of CI currently exposed to natural hazards. Existing regional studies have identified spatial patterns of infrastructure assets within a predefined region (e.g. segments along the coast)^23,24^, or have explored CI exposure to natural hazards by overlaying spatial hazard data with data on infrastructure assets at a national scale^25^. More advanced methodologies have incorporated data on interdependencies and user demand to identify CI hotspots^8,9,26^. At the larger European scale, the development of a harmonized dataset of CI^27^ has aided the assessment of multi-hazard risk^15^, and recently the very first estimates of multi-hazard risk to transport infrastructure were calculated at the global scale^6^.

Despite these advances, a global database integrating the geospatial locations of main CI systems is still lacking. In addition, there is currently no CI index to classify global hotspots of CI. Studies in other domains have demonstrated the value of using an index-based approach. For instance, the Social Vulnerability Index (SoVI)^28^ has been widely applied to assess the vulnerability of societies to natural hazards^29,30^. Another example is the Human Development Index (HDI), which is a composite measure representing levels of health, education and standard of living^30,31^. Recently, an index has been developed to indicate a value of infrastructure situated in predefined segments along the coastline of the UK^32,33^, using data on commercial and residential properties, among other things. However, this index is not exclusively focused on incorporating CI assets.

This study presents the first publicly available harmonized global spatial dataset for the representation of CI systems. As part of this dataset, we develop a first-of-its-kind composite index to express the spatial intensity of CI at the global scale, at a resolution of 0.10 × 0.10 and 0.25 × 0.25 degrees: the Critical Infrastructure Spatial Index (CISI). The CISI is expressed in a dimensionless value ranging between 0 (no CI intensity) and 1 (highest CI intensity). The index aggregates high resolution geospatial information on multiple CI assets per CI system. For the development of this index, we selected 39 CI types and categorized them under seven overarching CI systems: *transportation*, *energy*, *telecommunication*, *waste*, *water*, *education* and *health*. To validate the CISI, we compare it with subnational data on Gross Domestic Product (GDP) and population distribution.

The global dataset presented in this paper will be a valuable starting point for policy makers, planners, and researchers in several fields. The dataset can be deployed as a tool to gain insights in the current landscape of the CI network, to identify hotspots of CI, and to gain exposure information for risk assessments. In addition, the dataset can be used to reveal regions where additional efforts are needed to fill gaps in the mapping of infrastructure. We use open data hosted by OpenStreetMap (OSM), and provide code for further use and development (see Code Availability). In this study, we demonstrate the database and CISI at a global scale, and the open access code can also be used to further develop the dataset with latest releases of data on CI provided by OSM as well as other (open) sources for any location and any resolution.

## Methods

**Fig. 1:**
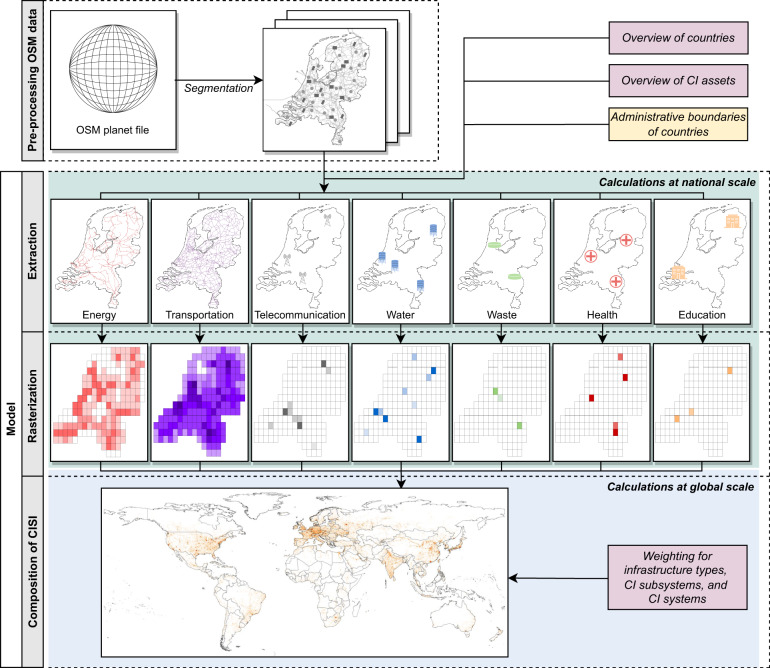
Schematic display of workflow. The green panel represents the part of the model that performs calculations at a national scale, and the blue panel represents the part of the model that performs calculations at a global scale. On the right-side, the purple-coloured boxes show the specifications required for the model. The yellow box indicates the spatial input required.

Figure 1 provides an overview of the methodology developed to create our harmonized global dataset. Each of the steps is described in detail in the following subsections. In brief, the methodology involves: (1) *pre-processing OSM data –* in this step, we disaggregate the global unprocessed OSM database to create an individual data file for each country; (2) *extraction* – in this step, we extract the geospatial location for a selection of CI; (3) *rasterization -* in this step, we develop a consistent rasterized dataset containing information on the amount of CI; and (4) the *composition of CISI* - in this step, we summarize the geospatial information from step 3 to calculate an index to express the spatial intensity of CI.

### Pre-processing of OSM data

Central to the development of the global dataset is the integration of open data collected and provided by OSM. The goal of this platform is to create and distribute free and openly accessible geospatial and attributional information on the world’s features. With 4.5 million map changes/day, the OSM database counts approximately 15.5 billion georeferenced features as of 26^th^ November 2020^34^.

**Fig. 2:**
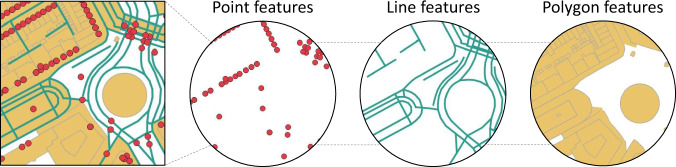
Visualization of raw OpenStreetMap data of a given area, with a breakdown by the datatypes.

Geographical features in OSM are projected in the form of *nodes*, *ways* and *relations*. A *node* represents a specific point in space and is defined by its latitude and longitude (e.g. telecommunication tower). The datatype *ways* exist as a line segment that is connected by two or more nodes (e.g. road). A polygon (or area) is described as *closed ways*. They are constructed from *ways* and created when the last node of a series of line segments is connected to the beginning (e.g. hospital). Another datatype, *relations*, is an ordered list of features that groups *nodes*, *ways* and *relations* into a larger unit. An example of unprocessed OSM data, including a breakdown of the basic datatypes, is shown in Fig. 2. Each georeferenced element in OSM has an id number that uniquely identifies it, and includes other details such as the user who modified the element and the time of last modification. Elements can be further specified by a list of attribute tags in the form of key-value pairs, whereby the value provides more detail to the key identifier. For example, primary roads that often link larger towns are specified under the key ‘highway’ in combination with the value ‘primary’.

The global OSM dataset containing all state-of-the-art mapped infrastructure is available via https://planet.openstreetmap.org/, which we downloaded on 8^th^ January 2021 in PBF format. Subsequently, the OSM planet file is disaggregated into smaller .PBF files at national level by using publicly available code^35^.

### Extraction of critical infrastructure

The second step is to extract all the unique CI assets from the OSM dataset. No clear guiding rules exist on which specific infrastructure assets can be prioritized as critical^36,37^, and the way the definition of CI is interpreted may vary per country. In this study, we represent the world’s infrastructure network by seven overarching CI systems: *transportation*, *energy*, *water*, *waste*, *telecommunication*, *education*, and *health*. This is in line with the classification of infrastructure systems discussed in the literature^1,2,8,21^, whereby infrastructure related to education and health has started to gain increasing attention recently^22^. We further subdivided these CI systems into a total of ten subsystems. Each subsystem contains two or more specific infrastructure types, for example the *telecom* subsystem contains infrastructure types *communication tower* and *mast*. For an overview of the classification of the seven CI systems, ten subsystems, and the selection of infrastructure types, refer to Section Data Records. From the list of active OSM key-value pairs^38^, we selected 81 OSM tags to represent 39 infrastructure types (see Supplementary Table 1 for the categorization of CI, and the reclassification).

The specified infrastructure types are extracted from the pre-processed OSM files at national level. We define ${\iota }_{t,xy}$ as a unique CI asset $\iota$ containing a specific set of *xy* coordinates, belonging to a specific infrastructure type *t*. We then define ${I}_{t,xy}=\left\{{\iota }_{t,1},\ldots ,{\iota }_{t,n}\right\}$as the set of all *n* CI assets of a specific infrastructure type. This may be, for instance, a set of CI assets that represents the infrastructure type *telecom towers*. Finally, we clip the set ${I}_{t}$ with administrative boundaries to ensure that we only capture CI assets that fall within the administrative boundaries of a country.

**Fig. 3:**
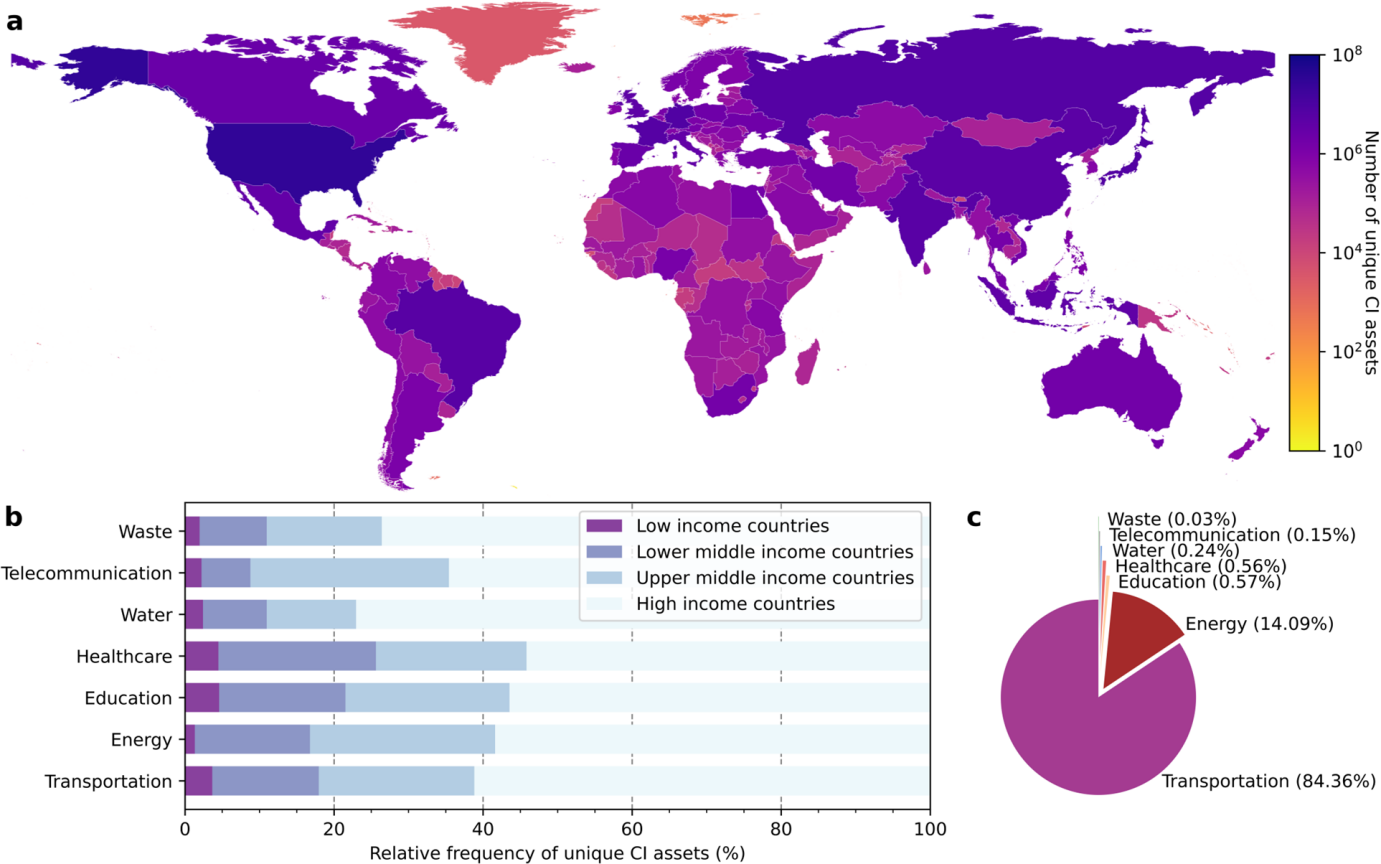
Distribution across space and statistics of the unique CI assets (point, line, and polygon data) that are returned after the extraction of OSM data. Panel (**a**) presents the number of unique CI assets per country. Panel (**b**) presents the percentage of unique CI assets for four income classes categorized by the main CI systems. Panel (**c**) presents the share of unique CI assets per main CI system.

The extraction of CI results in almost 153 million unique OSM elements. The data have a global coverage, with the highest number of unique CI assets found in the United States, followed by Germany and Japan (Fig. 3a). The lowest number of unique CI assets per country is mainly found in the Small Island Developing States (SIDS), and other small islands spread across the Atlantic and Pacific Ocean. Fig. 3b shows the number of unique elements per main CI system with a further specification by income class. Here, we identify a general pattern that holds for the seven main CI systems. Namely, the high-income countries have the largest share of unique CI assets for each CI system, whereas the low-income countries have the lowest contribution to this share. The high-income countries account for 60.8% of the extracted OSM elements, upper middle countries for 21.4%, lower middle countries for 14.5%, and the low-income countries for 3.3% (see details aggregated to the country level in Supplementary Table 2).

#### Transportation

The transportation system is sub-divided into three subsystems: roads, railways, and airports. The road network provided by OSM has a completeness-level of approximately 83% in January 2016^39^. We aggregate the 15 classes originally used by OSM to describe roadways to three classes: *primary, secondary* and *tertiary roads*. For the subsystem railways, we selected seven OSM key-value pairs that were aggregated to one common class (see Supplementary Table 1).

**Fig. 4:**
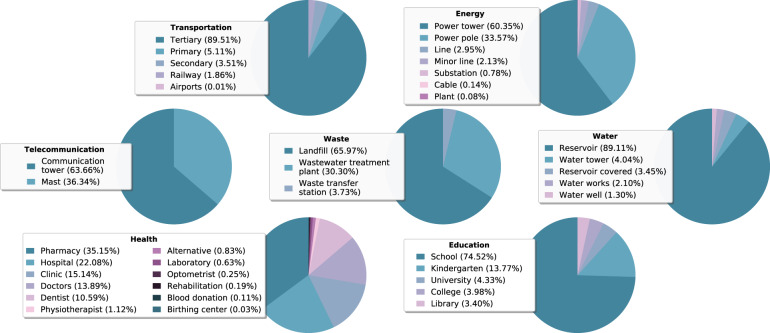
Relative number of unique CI assets extracted for the 39 infrastructure types, categorized by the seven main CI systems: transportation, energy, telecommunication, waste, water, education, and health.

The share of the number of unique elements belonging to the transportation system to the total number is dominant: 84% of the extracted CI elements belong to the transportation system (Fig. 3c). Fig. 4 provides more detail on the composition of the transportation system by highlighting the percentages of extracted unique assets per infrastructure type. Here, we find that the tertiary roads (90%) account for the most of unique assets. The total length of road infrastructure extracted from OSM is over 51 million kilometers, of which approximately 42.6 million is tertiary, 5.1 million primary, and 3.7 million secondary. We extracted over 2 million kilometers of railway infrastructure, and 17,508 airports worldwide.

It is worth noting that ports are not explicitly specified in this study, even though they serve as critical hubs of the transportation network. Multiple CI assets that we included in this research are assets that are typically situated in ports. Therefore, many of the CI assets of ports are captured, such as multiple road- and energy assets.

#### Energy

We selected seven infrastructure types for the representation of the energy system. These infrastructure types are related to the production, conversion and delivery of energy, and includes the following infrastructure types: *cable, line*, *minor line*, *power tower, power pole, plant*, and *substation*.

Cables are described by OSM as insulated assets that allow electrical power transmission or distribution in complex environments, such as indoors, underground, or undersea. In contrast, power lines are energy assets that are built above the surface and are usually carried by supporting structures. Here, OSM distinguishes between power lines that are supported by power towers, and minor power lines that are supported by poles used for low-voltage transmission. A power plant is an industrial, large-scale facility for the generation (or storing) of electricity. In general, a facility is tagged as a power plant if it generates more than 1 MW. Substations are used for the transmission and distribution of electricity within the energy network, whereby they transform high voltages to low voltages, or vice versa.

The composition of the energy system is presented in Fig. 4. In total, the dataset consists of 28,750 kilometers of power cables, over 4,3 million kilometers of power lines, and 571,416 minor lines. We find over 20 million supporting structures, of which 64% can be accounted for by power towers and the remaining 36% by power poles. The dataset contains 16,193 plants globally, and 167,190 substations.

#### Telecommunications

The telecommunications system is represented by two infrastructure types: *communication tower* and *mast*. We used a combination of three key-value pairs to extract these infrastructure types (see Supplementary Table 1). Communication towers are used for transmitting (a range of) radio applications (e.g. televisions, radio, and mobile phone), are often characterized by a height of over 100 meters, and are usually made of concrete. Masts, in contrast, are usually only used for a single application, and are a couple of meters high. Globally, the dataset counts approximately 141,478 communication towers and 80,750 masts (see Fig. 4).

#### Waste

For the waste system, we made a distinction between solid waste and water waste. Accordingly, the waste system is sub-divided into two subsystems. The solid waste subsystem is represented by infrastructure types *waste transfer station* and *landfill*. We represent the water waste subsystem using the infrastructure type *water waste treatment plant*. Solid waste is consolidated and transferred in bulk at waste transfer stations, whereas water waste is treated at water waste treatment plants. Landfills are sites for permanent or long-term storage of consolidated waste materials (that often come from waste transfer stations). We extracted 1,951 waste transfer stations, 34,551 land fill sites, and 15,870 water waste treatment plants at a global scale (see Fig. 4).

#### Water

The CI system water entails infrastructure that is critical for the water supply. We selected five infrastructure types that provide services for the extraction, distribution, and storage of both potable and non-potable water: *water tower*, *water well*, *reservoir covered*, *reservoir*, and *water works*. A water well is used to extract groundwater. Water works and water towers are both critical for the distribution of water. Here, water works are facilities that are used to apply water to the water pipe network, and water towers are elevated structures to pressurize the distribution network. OSM categorizes large man-made tanks for the storage of water as *reservoir covered*, whereas *reservoir* entails artificial lakes to store water. The extraction process resulted in a total of 370,218 unique water elements, with the infrastructure type *reservoir* having the highest contribution of 89%. Globally, we find 14,947 water towers, 4,801 water wells, 12,762 covered reservoirs, and 7,792 water works (see Fig. 4).

#### Education

The subsystem education is represented by five infrastructure types: *college*, *kindergarten*, *library*, *school*, and *university*. We extracted 863,928 education facilities from the OSM database. As is presented in Fig. 4, approximately 74.5% of the extracted education facilities are attributed to schools, followed by kindergartens (13.8%), universities (4.3%), colleges (4.0%), and libraries (3.4%).

#### Health

During the Ebola epidemic of 2014 in West Africa, a need arose for readily available information on the location of health facilities as well as specifics associated with a health facility (e.g. name of facility, number of doctors). As a result, the Global Healthsites Mapping Project (https://www.healthsites.io) has been launched with the aim to collect and validate a freely accessible global dataset on health facilities, which is being done in collaboration with OSM and other partners. Data on health facilities that are contributed via Healthsites.io are written to the OSM database, and vice versa. The types of health facilities included in this research are based on the list of health facilities that is defined by Global Healthsites and partners^40^. This list includes the following facilities: *doctor, pharmacy, hospital, clinic, dentist, physiotherapist, alternative, laboratory, optometrist, rehabilitation, blood donation, birthing center*.

We developed a procedure to include all georeferenced health facilities in a uniform way. Generally, multiple infrastructure types can be georeferenced as both point and polygon geometries. However, we found this inconsistency in georeferencing to be a substantial problem for the spatial completeness of health facilities. An examination of 16 randomly selected countries shed light on the usage of datatypes associated with the spatial completeness of the georeferenced health facilities. Only extracting health facilities as polygon geometries would exclude the health facilities that are exclusively tagged as point geometries, and vice versa. This reduces the spatial completeness of the health facility dataset.

The procedure entails the following steps. The set of facilities georeferenced as polygon data is merged with the set of facilities georeferenced as point data. However, prior to this, we check whether each polygon spatially intersects with point data in order to avoid double-counting. In case a spatial intersection exists, the polygon is only removed from the dataset if it concerns the same infrastructure type. This means that, for example, a specific hospital that is tagged as polygon geometry will only be removed from the dataset if: (1) it has a spatial intersection with a point feature; and (2) this point feature is tagged as a hospital. A filtered set of facilities georeferenced as polygon data remains, which is subsequently transformed into point geometries by taking the centroid of a polygon. Finally, this is merged with the set of facilities that are georeferenced as points in the original dataset. The number of health facilities at the global scale is 862,548. The composition of unique CI assets for the twelve infrastructure types defined for the CI system health is illustrated in Fig. 4.

### Rasterization of CI data

The next step is to translate the detailed geospatial information on CI into a consistent rasterized dataset, whereby each grid cell holds information on the estimated amount of infrastructure. We created a consistent raster of the globe with a resolution of 0.10 × 0.10 degrees, which is approximately 11.1 × 11.1 km at the equator, and a second raster with a resolution of 0.25 × 0.25 degrees. We spatially overlay all individual CI assets with each grid cell of the consistent raster of the globe. Each grid cell in this raster can be defined as a rectangle $p\left({x}_{1},{x}_{2},{y}_{1},{y}_{2}\right)$, and the collection of grid cells can be denoted by the set $P=\left\{{p}_{1},\ldots ,{p}_{z}\right\}$. The collection of unique CI assets of a specific infrastructure type within a given grid cell is denoted as ${I}_{t,xy}\left(p\right)=\left\{{\iota }_{t,1}\left(p\right),\ldots ,{\iota }_{t,n}(p)\right\}$, where $\left({x}_{1}\le x\le {x}_{2}\right)\wedge \left({y}_{1}\le y\le {y}_{2}\right)$. For example, this may be the collection of all unique telecom towers that are located within a given cell.

We use this collection of CI assets ${I}_{t}\left(p\right)$ to estimate the total amount of each infrastructure type within a given grid cell. The amount of infrastructure associated with one unique CI asset ${\iota }_{t,xy}$ is denoted as a number $\varphi \left({\iota }_{j}\right)$, where *j* reflects the datatype of the considered asset $\iota$, and the unit of spatial measurement is dependent on the datatype *j*. Depending on the datatype of an infrastructure type (see Supplementary Table 1), we used the following method to rasterize the global CI. We estimate: (1) the total count if the datatype *j* is a node; (2) the length in km is if the datatype *j* is a line; (3) and the area in km^2^ if the datatype *j* is a polygon. We can define the total amount of infrastructure for an infrastructure type in a given grid cell *p* as $s\left({I}_{t}\right)=\varphi \left({I}_{t}(p)\right)$. For example, this could be a given grid cell *p* that counts four telecom towers. The rasterized data per infrastructure type are then denoted as the set $S\left({I}_{t}\right)=\left\{\varphi \left({I}_{t}\left({p}_{1}\right)\right.,\ldots ,\varphi \left({I}_{t}\left({p}_{z}\right)\right.\right\}$. Using this procedure, the detailed geospatial information on the 39 selected infrastructure types is translated into two sets of 39 consistent rasterized layers containing geospatial information on the amount of infrastructure at a global scale (see Section Data Records).

### Composition of CISI

**Fig. 5:**
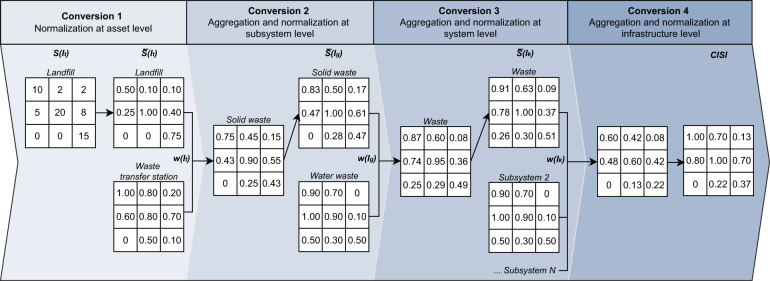
Schematic representation of the four conversions applied to derive the CISI. The procedure is illustrated for one branch of the CI dataset, starting from landfill assets up to the aggregation of CISI.

The final step is to develop the CISI, which is a spatial composite of the rasterized data per infrastructure type. For the development of CISI, a four-fold conversion is needed, which is summarized in Fig. 5, and can be described as follows.

*Conversion 1*: We normalize the 39 consistent rasterized layers at asset level that are described in Subsection Rasterization of CI data. The normalization of rasterized data is a prerequisite to enable comparison between the different infrastructure types, but also to ensure comparison between multiple datatypes. To normalize each of the 39 rasterized layers representing a given infrastructure type $S({I}_{t})$, we first detect the grid cell containing the highest amount of infrastructure, whereby the amount of infrastructure in this specific grid cell is denoted as $\alpha ={\rm{\max }}\left(S\left({I}_{t}\right)\right)$. Subsequently, each grid cell of a given rasterized layer representing a given infrastructure type $S({I}_{t})$ is divided by the highest amount of infrastructure *α*, resulting in a normalized layer $\bar{S}\left({I}_{t}\right)$. For example, for the rasterized layer containing information on global landfills (shown in the first panel of Fig. 5), we detect that the grid cell with the highest amount of infrastructure holds 20 km^2^ of land fill assets. Subsequently, all of the grid cells in the rasterized layer are divided by 20. The first conversion results in a dimensionless value ranging between 0 (no landfill assets) and 1 (highest intensity of landfill assets). The procedure for the normalization at asset level is described by Eq. 1:

1 $$\bar{S}\left({I}_{t}\right)=\frac{S({I}_{t})}{\propto }\quad \quad {\rm{with}}\propto ={\rm{\max }}\left(S\left({I}_{t}\right)\right)$$

*Conversion 2*: We aggregate the 39 normalized layers at infrastructure asset level into ten normalized layers at subsystem level. An infrastructure type *t* belongs to a specific subsystem *g*. Accordingly, the normalized layers for infrastructure types within a given subsystem are combined into an aggregated layer at subsystem level $\bar{S}\left({I}_{g}\right)$, which represents the spatial intensity of that specific subsystem. The number of infrastructure types *T* within a subsystem *g* is denoted as ${T}^{g}$. To continue with the example provided in Fig. 5, the solid waste subsystem is represented by two infrastructure types ${T}^{g}$, namely landfill and waste transfer station. The normalized layers for these infrastructure types are combined into an aggregated layer representing the solid waste subsystem. We use an equal weighting, which means that each infrastructure type is considered equally as important. We denote the weighting of a given infrastructure type as $w({I}_{t})$. The product of the summation, denoted as ${\sum }_{t=1}^{\left|{T}^{g}\right|}\bar{S}\left({I}_{t}\right)\ast w\left({I}_{t}\right)$, is normalized using the same method as in the first conversion. The second conversion is expressed by Eq. 2:

2 $$\bar{S}({I}_{g})=\frac{{\sum }_{t=1}^{| {T}^{g}| }\bar{S}({I}_{t})\ast w({I}_{t})}{{\rm{\max }}({\sum }_{t=1}^{| {T}^{g}| }\bar{S}({I}_{t})\ast w({I}_{t}))}\quad {\rm{with}}\;t\in g$$

*Conversion 3*: We aggregate the geospatial data per subsystem into seven layers at system level. This conversion is similar to the previous step, but applied at system level. A given subsystem *g* is categorized under a specific system *k*, whereby the total number of subsystems belonging to a system *k* is expressed by ${G}^{k}$. We aggregate the geospatial information of the subsystems, in additive format with equal weighting, where the weighting for a given subsystem is denoted as $w({I}_{g})$. This is then followed by a normalization to derive $\bar{S}\left({I}_{k}\right)$. For example, the water waste subsystem and the solid waste subsystem comprise the waste system (Fig. 5). In this step, the two subsystems are combined to develop a normalized layer at system level, representing the spatial intensity of the overall waste system. We denote the third conversion as Eq. 3:

3 $$\bar{S}\left({I}_{k}\right)=\frac{{\sum }_{g=1}^{{G}^{k}}\bar{S}\left({I}_{g}\right)\ast w\left({I}_{g}\right)}{{\rm{\max }}({\sum }_{g=1}^{{G}^{k}}\bar{S}\left({I}_{g}\right)\ast w\left({I}_{g}\right))}\quad {\rm{with}}\;g\in k$$

*Conversion 4*: The CISI is developed in the final conversion. The CISI is the aggregation of the CI systems *K* representing the global infrastructure *c*. Here, the total number of CI systems is expressed by ${K}^{c}$. The composition is again based on equal weighting, denoted as $w\left({I}_{k}\right)$ for a given system *k*, followed by a normalization. The last step is represented by Eq. 4:

4 $$CISI=\bar{S}({I}_{c})=\frac{{\sum }_{k=1}^{{K}^{c}}\bar{S}({I}_{k})\ast w({I}_{k})}{{\rm{\max }}({\sum }_{k=1}^{{K}^{c}}\bar{S}({I}_{k})\ast w({I}_{k}))}\quad {\rm{with}}\;k\in c$$

We execute conversion 1–4 to derive the CISI at the global scale for two resolutions (0.10 × 0.10 and 0.25 × 0.25 degrees). As mentioned earlier, an equal weighting is applied in this paper for the aggregation of the components at infrastructure type, sub-system and system level. Yet we would like to stress that the developed model allows for adjustments of the weightings of the infrastructure types, sub-systems, and systems. This means that a user can, for example, increase the weight of power stations to emphasize the importance of this infrastructure type to society. The adjustment of weights can be done on the basis of expert judgement or extensive literature reviews, allowing for a tailored CISI dataset with adjusted weightings that meets the specific need of the end user. However, developing the different weightings for the various components is not within the reach of this study, and indeed the weightings will differ depending on the context of the study in which the data are used.

**Fig. 6:**
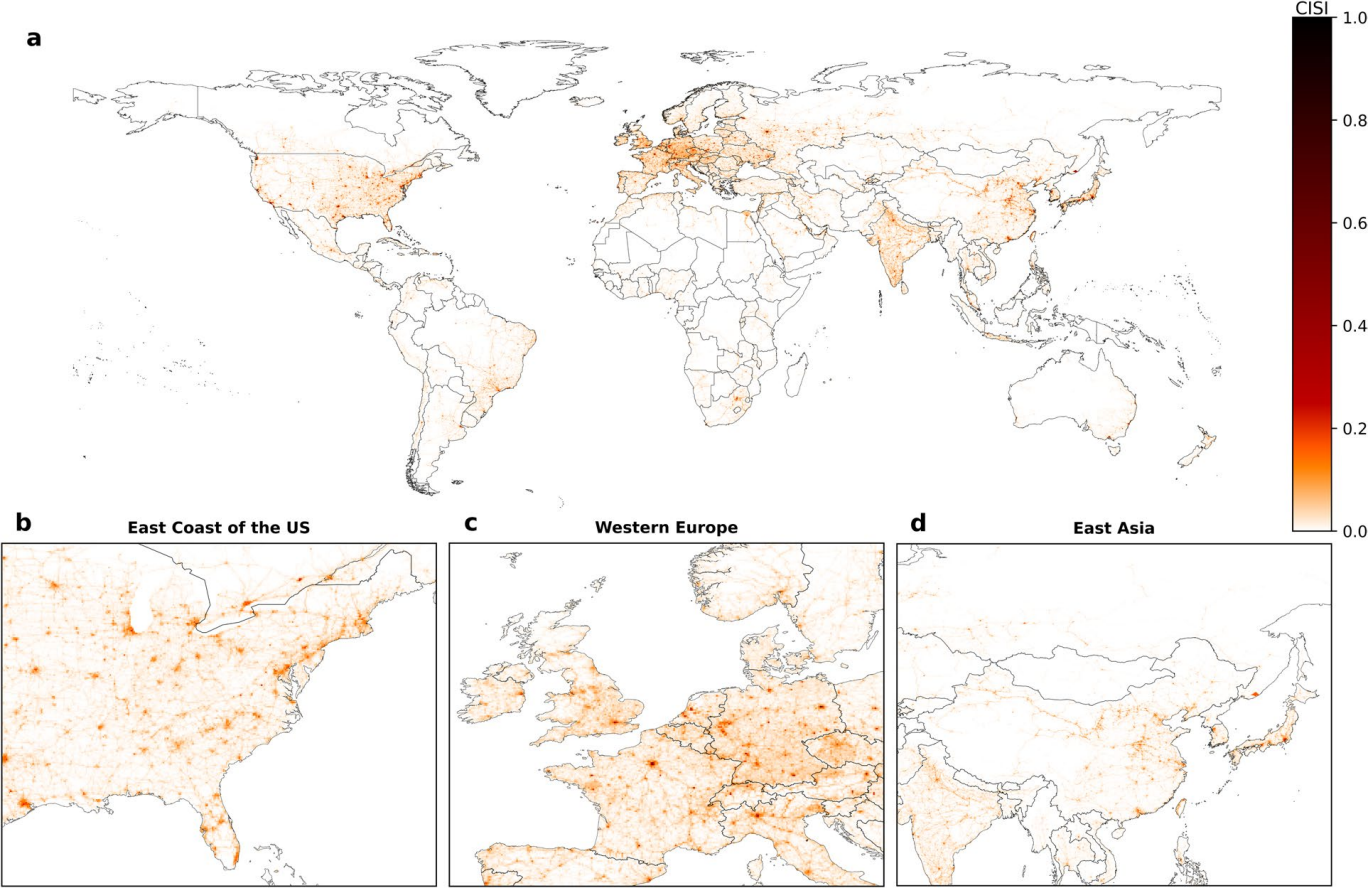
Global visualization of the Critical Infrastructure Spatial Index (CISI) at a resolution of 0.10 × 0.10 degrees. Panel (**a**) represents the CISI at the global scale, whereas panel (**b**–**d**) provides more detail at regional level for the East Coast of the US (**b**), Western Europe (**c**), and East Asia (**d**).

The result of the CISI at the global scale with a resolution of 0.10 × 0.10 degrees are presented in Fig. 6., highlighting the disparities between areas where high amounts of CI are located and where not. The CISI ranges between 0 (no CI) and 1 (highest CI intensity). The CISI normalized at the global scale gives valuable information on where certain amounts of infrastructure are located. However, we would like to emphasize that locations with less amounts of infrastructure are not lot of less importance to society. In addition to this dataset, we therefore also execute conversion 1–4 at the continental scale. The datasets at continental scale allow for the comparison of the CI intensity, and thus amounts of infrastructure, across continents in a relative way.

## Data Records

The spatially-explicit harmonized global dataset of CI is publicly available from the Zenodo repository^41^. This is provided in standard WGS84 coordinate system at multiple resolutions: 0.10 × 0.10 and 0.25 × 0.25 degrees. Tables 1 and 2 give an overview of the outputs that are part of the global CI dataset.

**Table 1:** List of infrastructure types considered in this study, categorized under ten subsystems and seven systems. Also, an overview of names of the output files containing information on the amount of infrastructure is provided.

System	Subsystem	Infrastructure type	Raster filename	
Energy	Power	Cable	summary_energy.feather	cable.tif
		Line		line.tif
		Minor line		minor_line.tif
		Plant		plant.tif
		Substation		substation.tif
		Power tower		power_tower.tif
		Power pole		power_pole.tif
Transportation	Railways	Railway	summary_transportation.feather	railway.tif
	Roads	Primary		primary.tif
		Secondary		secondary.tif
		Tertiary		tertiary.tif
	Airports	Airport		airports.tif
Telecommunication	Telecom	Communication tower	summary_telecommunication.feather	communication_tower.tif
		Mast		mast.tif
Water	Water supply	Water tower	summary_water.feather	water_tower.tif
		Water well		water_well.tif
		Reservoir covered		reservoir_covered.tif
		Water works		water_works.tif
		Reservoir		reservoir.tif
Waste	Solid waste	Landfill	summary_waste.feather	landfill.tif
		Waste transfer station		waste_transfer_station.tif
	Water waste	Water waste treatment plant		wastewater_treatment_plant.tif
Health	Healthcare	Clinic	summary_healthcare.feather	clinic.tif
		Doctors		doctors.tif
		Hospital		hospitals.tif
		Dentist		dentist.tif
		Pharmacy		pharmacy.tif
		Physiotherapist		physiotherapist.tif
		Alternative		alternative.tif
		Laboratory		laboratory.tif
		Optometrist		optometrist.tif
		Rehabilitation		rehabilitation.tif
		Blood donation		blood_donation.tif
		Birthing center		birthing_center.tif
Education	Education	College	summary_education.feather	college.tif
		Kindergarten		kindergarten.tif
		Library		library.tif
		School		school.tif
		University		university.tif

**Table 2:** Overview of names of the output files containing information on the CISI.

Critical Infrastructure Spatial Index	Raster filename	
Africa	CISI_africa.feather	CISI_ africa.tif
Asia	CISI_asia.feather	CISI_ asia.tif
Europe	CISI_europe.feather	CISI_europe.tif
Oceania	CISI_oceania.feather	CISI_oceania.tif
Central-America	CISI_central_america.feather	CISI_central_america.tif
North-America	CISI_north_america.feather	CISI_north_america.tif
South-America	CISI_south_america.feather	CISI_south_america.tif
Global	CISI_global.feather	CISI_global.tif

For both resolutions, geospatial information on the estimated amount of infrastructure for each infrastructure type is stored in GeoTIFF format, resulting in 39 .tif files that can be used for infrastructure type-specific analysis (Table 1). These files can be easily accessed, visualized, and manipulated using appropriate GIS applications. In addition to this, seven files in feather format are provided^42^, containing the geospatial information on the estimated amount of infrastructure per system. This feather format has been chosen over geopackages because of advantages in increased writing and reading speed. Within these feather files, geospatial information is stored as Well-Known-Binary (WKB).

Lastly, feather and GeoTIFF files containing spatial information on the CISI and the sub-scores per system are provided. Here, the CISI is a dimensionless proxy scaled between 0 (no CI intensity) and 1 (highest CI intensity) as a result of the composite index procedure described in the Methods Section. We provide the CISI normalized at global level, and per continent (Table 2). Again, these files are provided for the two resolutions.

## Technical Validation

Measuring and improving the quality of OSM data has been an ongoing challenge. In response, researchers have increasingly focused on the development of tools to support quality assessments both in terms of extrinsic quality approaches (i.e. comparison to external reference datasets), and intrinsic approaches (i.e. assessing history information of OSM)^43^. Recent developments include approaches to predict completeness of building footprints using remote sensing data^44^, and the application of conventional neural networks (CNN) to improve the spatial accuracy of buildings in rural areas^45^. As momentum has been created to progress the reliability of OSM, and tools are continuously being developed, the emphasis of this study is rather on the application of OSM data than on seeking a dataset with complete spatial coverage. Therefore, a systematic validation of each selected infrastructure type to assess the quality of OSM data at the global scale is out of the scope of this study. However, we provide information on the reliability of the composite of infrastructure types - the CISI.

The CISI is a first-of-its-kind dataset, and therefore it is not possible to execute a validation exercise by means of comparing a reference dataset with the same metrics. Accordingly, we designed an approach to assess the validity of the CISI by means of the evaluation of the plausibility of the spatial distribution of the CISI values. For the validation of the CISI, we correlate the CISI dataset with two socio-economic parameters: GDP and population distribution. The spatial pattern of population distribution can be used as a proxy to indicate built environments^46^, therefore also indicating built-up areas where infrastructure types, that comprise the CISI, are located. Infrastructure contributes to socio-economic development^1,47^, of which GDP is a key indicator^48^. The relation between infrastructure and GDP has been used for improved mapping of regional GDP^49^, and to evaluate the feasibility to predict the level of socio-economic development^50,51^.

We use high-resolution gridded population distribution and GDP open source data for the validation procedure. WorldPop provides yearly global estimations on the population counts since 2000. We select the latest dataset^52^, which contains the total number of people per grid cell of 30 × 30 arc-second (1 × 1 km at equator) in 2020 derived by top-down modelling approaches. Kummu *et al*.^48^ have developed GDP global annual gridded datasets for the 25-year period of 1990–2015. GDP is derived from a combination of sub-national and national datasets, and population data. We select the dataset^53^ containing the average GDP, expressed in International US dollars, per grid cell of 30 arc-second in 2015. We calculate both the population count and GDP per CISI grid cell, and then use the Spearman’s rank-order correlation to provide the Spearman’s correlation coefficient ${r}_{s}$ at a global scale, and per continent for better insight into spatial variations.

The results of the validation are reported in Table 3. The CISI is positively correlated with both GDP and population count; high levels of GDP and population correlate with high levels of CI intensity. The results show that the correlation coefficients obtained for GDP and population count are of the same magnitude. The CISI dataset at the global scale with a resolution of 0.10 × 0.10 degrees shows a correlation coefficient of approximately 0.70 for both GDP and population. The obtained correlation coefficients are higher for the 0.25 × 0.25 degrees global dataset with a ${r}_{s}$ of 0.75 and 0.77 for GDP and population count, respectively. However, we find variations in the correlation coefficients derived at continental scale. As expected, the highest correlation coefficients are obtained for North America and Europe, while the correlation coefficients obtained for Africa are the lowest.

**Table 3:** Overview of the Spearman’s Rank Correlation Coefficients for the CISI datasets at global and continental scale with GDP and population distribution (p < 0.001).

Critical Infrastructure Spatial Index	Resolution: 0.10 × 0.10 degrees		Resolution: 0.25 × 0.25 degrees	
	Spearman’s Rank Correlation Coefficient		Spearman’s Rank Correlation Coefficient	
	GDP	Population	GDP	Population
Africa	0.523	0.591	0.594	0.646
Asia	0.624	0.725	0.717	0.760
Europe	0.742	0.835	0.808	0.885
Oceania	0.733	0.610	0.722	0.646
Central America	0.699	0.746	0.810	0.800
North America	0.819	0.838	0.848	0.882
South America	0.634	0.649	0.704	0.732
Global	0.699	0.694	0.748	0.770

The findings of this validation procedure should be viewed as a step forward, and establish new grounds for in-depth analysis. For example, extensive analysis is required to fully understand how each infrastructure type is correlated with GDP and population distribution. The dataset can be used for various purposes (see Usage Notes), and therefore different follow-up actions arise depending on usage. For instance, this validation procedure paves the way for risk modellers to better understand to what extent OSM data can be used for risk assessments, and whether they should consider complementary data (for specific areas). On the other hand, the validation procedure described in this section can support the OSM mapping community to target under-mapped areas, and even to specify which infrastructure types are underrepresented in OSM.

## Usage Notes

The harmonized global spatial dataset for the representation of CI has the potential to support a wide range of applications, with the overall aim to work towards robust and resilient CI for the global population that is able to cope with current and future threats. Accordingly, this dataset can support indicators for the global progress in achieving global goals and frameworks such as the SDGs and the SFDRR. We provide examples to illustrate potential usage of the dataset.

The SFDRR, for example, defines multiple indicators, such as damage to CI, and the number of destroyed or damaged facilities attributed to disasters^54^, to ‘substantially reduce disaster damage to critical infrastructure and disruption of basic services … by 2030’^22^. Valuable information that can contribute to this goal, either in the defined indicators or in newly developed metrics, can be retrieved by incorporating the presented dataset for risk assessments. The risk for natural hazards is often identified by combining information on three components: hazard, exposure and vulnerability^6,15,55^. The dataset developed here provides a high-level overview of global infrastructure, which, for example, can be used for exposure hotspot analysis, whereby an overlay is made with information on the extent and magnitude of a given natural hazard (see for example Dilley *et al*.^56^). By including the vulnerability component that expresses the susceptibility of infrastructure to a hazard, the risk can be estimated for regions. As aforementioned, the CISI is the aggregation of various infrastructure types expressed in a dimensionless quantity representing the amount of CI in relative proportions between grids. Therefore, the final risk metric may be in the form of a risk index such as the one presented by Izaguirre *et al*.^57^. In addition, as explained in Data Records Section, we also provide the gridded amount of infrastructure per infrastructure type in absolute values, which can be used to determine the amount of infrastructure under risk (e.g. number of hospitals).

Hence, the gridded harmonized global dataset for CI has the potential to be used for rapid high-level spatial risk assessments to identify both areas and types of infrastructure that require in-depth analysis. This could indicate hotspot locations in which a more in-depth analysis may be required using infrastructure data in object format. Such object format data can be extracted from OSM using the code developed for this study (see Code availability for details). Insights from these assessments are fundamental to effectively prioritize areas under risk and for strategic planning.

Also, the dataset can be deployed as a tool to gain insights in the current landscape of global CI. Large parts of the global population still lack coverage of essential services: the United Nations estimates that 789 million people have no access to electricity; 2.2 billion have no access to safely managed drinking water; and more than half of the global population has no access to essential health services^58^. Here, the dataset can be applied as a proxy to indicate regions without or with low amounts of infrastructure, in combination with, for instance, population data. Such efforts are needed as infrastructure is directly or indirectly relevant for the achievement of the 17 SDGs^21^, and to prioritize where investments in CI are most crucial. The dataset also allows for the identification of CI hotspots for specific infrastructure types and for the overall CI network.

When using the dataset, users should be aware that the presented dataset does not have a complete spatial coverage, simply because of the incompleteness of OSM. On the one hand, this is expected to result in an underestimation of the risk when applying the dataset for risk assessments. On the other hand, this may result in an overestimation of indicated regions lacking infrastructure. The CISI and the rasterized layers per infrastructure type, however, have potential to identify areas that need additional mapping efforts. For example, on country level, we find that Curacao has no mapped assets comprising the energy system, while we do expect power lines and other power infrastructure in this area to support other infrastructure and facilities (see Supplementary Table 2). Furthermore, this dataset does not account for complexities in interdependencies (see for example Thacker *et al*.^9^) as this is considered to be out of the scope for this study.

The model is built with great flexibility, and with the capability to process large amounts of high-detailed geospatial data at a global scale. Firstly, the dataset can easily be updated over time with the latest changes in OSM. Secondly, CI is not limited to the overview of CI infrastructure types selected for this research, and the associated categorizations. Therefore, the model can be expanded to any number of infrastructure types, subsystems, and CI systems. Thirdly, we applied an equal weighting to derive the CISI. However, the model allows for adjustments to the weightings, making it possible to enhance or diminish the importance of infrastructure types, sub-systems and CI systems. Fourthly, the code can also be used to create a harmonized dataset for smaller areas, and with any appropriate and suitable resolution that fits the need. Lastly, other sources offering geospatial data on CI, at any scale, can be added to the model. Users are encouraged to expand the current dataset by including other (freely accessible) sources.

## Supplementary information

Supplementary Table 1

Supplementary Table 2

## Data Availability

The code developed to process the OSM data is publicly available through the following GitHub repository: https://github.com/snirandjan/CISI^59^. The procedure for the developed CI dataset can be simulated using the main script, which is divided into three sections: (1) extraction of CI from OSM files in .PBF format, and reclassification; (2) estimation of amount of CI; and (3) calculation of the CISI. We also provide code for the validation procedure, and for the development of the figures and supplementary files. Detailed information per section and on the applied functions can be found on the repository, README file, and throughout the code.
